# Microsatellite genotyping of medieval cattle from central Italy suggests an old origin of Chianina and Romagnola cattle

**DOI:** 10.3389/fgene.2015.00068

**Published:** 2015-03-04

**Authors:** Maria Gargani, Lorraine Pariset, Johannes A. Lenstra, Elisabetta De Minicis, European Cattle Genetic Diversity Consortium, Alessio Valentini

**Affiliations:** ^1^Department for Innovation in Biological, Agro-food and Forest systems, University of TusciaViterbo, Italy; ^2^Faculty of Veterinary Medicine, Utrecht UniversityUtrecht, Netherlands; ^3^Department of Sciences of Cultural Heritage (DISBEC), University of TusciaViterbo, Italy

**Keywords:** microsatellite, ancient DNA, cattle, Ferento, NeighborNet

## Abstract

Analysis of DNA from archeological remains is a valuable tool to interpret the history of ancient animal populations. So far most studies of ancient DNA target mitochondrial DNA (mtDNA), which reveals maternal lineages, but only partially the relationships of current breeds and ancient populations. In this study we explore the feasibility of nuclear DNA analysis. DNA was extracted from 1000-years old cattle bone collected from Ferento, an archeological site in central Italy. Amplification of 15 microsatellite FAO-recommended markers with PCR products yielded genotypes for four markers. Expected heterozygosity was comparable with values of modern breeds, but observed heterozygosity was underestimated due to allelic loss. Genetic distances suggested a position intermediate between (1) Anatolian, Balkan, Sicilian and South-Italian cattle and (2) the Iberian, North-European and Central-European cattle, but also a clear relationship with two central-Italian breeds, Chianina and Romagnola. This suggests that these breeds are derived from medieval cattle living in the same area. Our results illustrate the potential of ancient DNA for reconstructing the history of local cattle husbandry.

## Introduction

The study of ancient DNA (aDNA) has developed since about 28 years from the analysis of short segments of mitochondrial DNA (mtDNA) to the spectacular whole-genome analysis of close relatives of the *Homo sapiens* (Higuchi et al., 1984; Pääbo, 1985; Reich et al., 2010). It complements archeological and historical investigations by providing ancestral links between ancient samples and present organisms (Vernesi et al., 2004; Caramelli et al., 2007). The majority of aDNA studies still target mtDNA, which is facilitated by its high copy number per cell. This allowed to determine the sequences of mtDNA control regions extracted from cattle remains excavated at several sites (Lenstra et al., 2014), but also the entire mitochondrial genome of two *Bos primigenius* specimens (Edwards et al., 2010; Lari et al., 2011). However, the genealogy inferred from the mtDNA not necessarily reflect that inferred with nuclear markers. Analysis of nuclear DNA from ancient remains is more challenging, but has been reported in several studies (Greenwood et al., 1999, 2001; Noonan et al., 2005; Pariset et al., 2007; Green et al., 2010; Reich et al., 2010). Ancient genotypes of nuclear microsatellites markers are informative for genetic diversity, subdivision and geographical origin (Edwards et al., 2003; Allentoft et al., 2011; Ishida et al., 2012; Nyström et al., 2012). Moreover, there are several studies carried out with the so called FAO list of microsatellites in almost every continent on a huge amount of individuals of several breeds (Ajmone-Marsan and The GLOBALDIV Consortium, 2010). This valuable database could be related to ancient remains in order i. to discover the closeness of ancient individuals to extant populations to understand the type of husbandry and production of the time and ii. to infer the origin, migration and admixture of present breeds.

Authentic cattle breeds in central and southern Italy are of the Podolian type (Felius, 1995). However, it is not yet clear if these are of ancient origin or have been imported (Felius et al., 2014). The aim of this study was to gain insight into history of Italian cattle by comparing 1000-year old cattle DNA of Italian origin with DNA from modern cattle. DNA was extracted from bones collected in Ferento, an archeological site near Viterbo in central Italy inhabited since Bronze Age, but developed mainly during Roman and Medieval ages. It rises on a triangular plateau made of tuff stone [Pianicara, IGM F.137 II NE] and delimited by two ditches: Guzzarella (or Vezzarella) and Acqua Rossa, subaffluents of the Tiber. The archeological investigations in Ferento revealed five areas, named assay I, assay II, assay III, assay IV, and assay V. The assay I comprises the area northward the Decumano in the medieval quarter; the assay II is localized on west area at the limit of the plateau, where an important necropolis was found; the assay III was occupied with a residential building on the southern side of the Decumano and with an important system of tanks, leant against the Theater; the assay IV, placed in the northwestern area of the plateau, and the assay V, near the assay I close to the fortification.

We compared the microsatellite genotypes obtained for four microsatellites with those of modern cattle breeds in order to uncover the origins of the Central-Italian medieval cattle.

## Materials and methods

### Ancient and modern samples

Thirty bone samples of medieval cattle (~1000 BP) were collected at Ferento, an archeological site near Viterbo in central Italy (Table 1). The analyzed remains were recovered from five different excavation areas. The dating was made on the basis of pottery found in the same layer. Moreover, one samples were sent to CEDAD (CEntre for DAting and Diagnostics) belonging to the University of Salento (Department of Innovation Engineering) and were carbon-dated using high resolution mass spectrometry. The results confirmed the age estimated by pottery layers. Species identification, for the selection of the samples to be analyzed, was based on morphology and dimensions of the specimens.

**Table 1:** **Medieval samples**.

**Sample**	**Element**	**Age**	**Excavation areas**	**GPS**	**Amplification**
Ferento 1	Astragalus	~1000 BP	5	42°29′21.2″N, 12°07′55.8″E	No
Ferento 2	Phalanx II	~1000 BP	3	42°29′18.4″N, 12°07′53.9″E	Yes
Ferento 3	Phalanx I	~1000 BP	4	42°29′19.2″N, 12°07′52.4″E	No
Ferento 4	Heel	~1000 BP	4	42°29′19.2″N, 12°07′52.4″E	Yes
Ferento 5	Molar 3	~1000 BP	3	42°29′18.4″N, 12°07′53.9″E	Yes
Ferento 6	Phalanx I	~1000 BP	4	42°29′19.2″N, 12°07′52.4″E	Yes
Ferento 7	Astragalus	~1000 BP	4	42°29′19.2″N, 12°07′52.4″E	Yes
Ferento 8	Astragalus sx	~1000 BP	4	42°29′19.2″N, 12°07′52.4″E	Yes
Ferento 9	Jawbone	~1000 BP	3	42°29′18.4″N, 12°07′53.9″E	No
Ferento 10	Phalanx II	~1000 BP	4	42°29′19.2″N, 12°07′52.4″E	Yes
Ferento 11	Jawbone	~1000 BP	4	42°29′19.2″N, 12°07′52.4″E	Yes
Ferento 12	Heel	~1000 BP	4	42°29′19.2″N, 12°07′52.4″E	No
Ferento 13	Jawbone	~1000 BP	2	42°29′19.4″N, 12°07′47.2″E	Yes
Ferento 14	Metatarsus	~1000 BP	3	42°29′18.4″N, 12°07′53.9″E	No
Ferento 15	Heel	~1000 BP	5	42°29′21.2″N, 12°07′55.8″E	Yes
Ferento 16	Phalanx I	~1000 BP	4	42°29′19.2″N, 12°07′52.4″E	No
Ferento 17	Astragalus	~1000 BP	1	42°29′20.6″N, 12°07′56.7″E	No
Ferento 18	Phalanx I	~1000 BP	1	42°29′20.6″N, 12°07′56.7″E	Yes
Ferento 19	Phalanx I	~1000 BP	1	42°29′20.6″N, 12°07′56.7″E	No
Ferento 20	Metatarsus	~1000 BP	4	42°29′19.2″N, 12°07′52.4″E	Yes
Ferento 21	Phalanx I	~1000 BP	4	42°29′19.2″N, 12°07′52.4″E	Yes
Ferento 22	Metatarsus	~1000 BP	4	42°29′19.2″N, 12°07′52.4″E	Yes
Ferento 23	Phalanx II	~1000 BP	3	42°29′18.4″N, 12°07′53.9″E	Yes
Ferento 24	Heel	~1000 BP	5	42°29′21.2″N, 12°07′55.8″E	Yes
Ferento 25	Metatarsus	~1000 BP	2	42°29′19.4″N, 12°07′47.2″E	No
Ferento 26	Phalanx I	~1000 BP	3	42°29′18.4″N, 12°07′53.9″E	No
Ferento 27	Humerus	~1000 BP	2	42°29′19.4″N, 12°07′47.2″E	No
Ferento 28	Phalanx II	~1000 BP	1	42°29′20.6″N, 12°07′56.7″E	No
Ferento 29	Phalanx I	~1000 BP	2	42°29′19.4″N, 12°07′47.2″E	No
Ferento 30	Metatarsus	~1000 BP	4	42°29′19.2″N, 12°07′52.4″E	No

In order to avoid contamination and degradation by sudden change in environmental conditions, samples were picked up using latex gloves, put immediately in vacuum-pack, stored at −20°C (Pariset et al., 2007) and transferred to a lab dedicated to ancient DNA analysis. In a dedicated laboratory room, the outer surface was removed with sand paper and bone powder was collected by drilling. DNA was extracted according to Yang et al. (1998) and quantified using a DTX Multimode Detector 880 (Beckman) with Picogreen method (Quant-iT PicoGreen, Invitrogen). At least two independent DNA extractions were performed. One single sample per day was extracted with a negative control. Comprehensive datasets of modern samples of genotypes of cattle breeds from Europe, Africa and Asia (Table S1) have been described previously (European Cattle Genetic Diversity Consortium, 2006; Medugorac et al., 2009; Laloë et al., 2010; Felius et al., 2011; Delgado et al., 2012).

### Microsatellite genotyping

Fifteen microsatellites were selected from the FAO list of recommended microsatellites (Table S2) (FAO, 2011). Only for three of these markers the published primers generated PCR products shorter than 160 bp. In order to optimize the amplification with aDNA templates, we redesigned primers for the other 12 microsatellites (Table S2) using Primer3 software. In a separate laboratory room, a first-round of PCR was carried out using a 20 μl reaction volume containing PCR buffer with 2.5 mM MgCl_2_, 200 μM of each dNTPs, 0.05 μM of both the forward (fluorescent labeled dyes) and the reverse primer (Proligo), 0.2 units of Bio-x-act short Taq Polymerase (Bioline) and 10 ng of DNA. A 5 min denaturation step at 94°C was followed by 30 cycles of 30 s denaturation at 94°C, 1 min at the respective annealing temperatures (Table S2) and 1 min of extension at 68°C, followed by a final 5 min extension step at 68°C. Products were purified with exosap (Promega). A second round of PCR was performed with as template the product (1 μl) of the first PCR reaction. During each PCR experiment both extraction and the PCR negative controls were checked for the absence of amplification products. At least four amplifications per sample were performed in order to monitor for allelic drop out. PCR products were separated by electrophoresis on a CEQ 8800 sequencer (Beckman Coulter) and sized with the standard CEQ™ DNA Size Standard Kit—400 (Beckman Coulter). The alleles were scored using the proprietary CEQ fragment analysis software.

Microsatellite dataset from modern cattle breeds were obtained from the EU Resgen project. In order to compare the allele lengths of the ancient cattle with those of the modern cattle database, we have included in all plates subject to PCR three modern samples (ITROM10, ITROM11, ITROM20) as a reference. The ancient cattle alleles were recalculated for comparison with the reference individuals. Genotypes were scored as heterozygous when the electropherogram showed a clear biallelic profile or when two different mono-allelic profiles were observed in different PCRs (Allentoft et al., 2011). The allelic profiles have been deposited in the Dryad database (http://dx.doi.org/10.5061/dryad.d4500). Allele frequencies and observed and expected heterozygosity were calculated for each population using MSToolkit. Genetic distances were calculated by using the program MSAT2 (http://genetics.stanford.edu/hpgl/projects/microsat/) and visualized in NeighborNet graphs with SplitsTree4 program (http://www.splitstree.org/). The NeighborNet graphs were simplified by combining several breeds in regional pools as approximations of ancestral gene populations. Thus, we made metapopulations of the dairy breeds from the Northwest-European Lowland, Central Brown cattle (including Swiss-Brown, Rendena, Bruna, and Cabannina), a West-Central cluster (Simmental and its derivatives); Iberian cattle, Sicilian Modicana and Cinesara, Balkan Busha, Anatolian cattle and Indopakistan zebu breeds, respectively. Principal component analysis of a matrix of F_*ST*_ genetic distances was performed using the program Genalex (Peakall and Smouse, 2006). Unsupervised model-based clustering was done with the program Structure (Pritchard et al., 2000).

## Results

Extraction of genomic DNA yielded for all 30 ancient samples quantifiable DNA and for 16 of these also amplification products. From the 15 microsatellites, four (INRA5, HEL1, MM12, CSRM60) could be amplified successfully in at least one sample. A total of 760 amplifications were performed on ancient samples. The success rate PCR per sample and per locus is shown in Table S3. Markers INRA5, HEL1, MM12, and CSRM60 yielded genotypes in 6, 8, 10, and 10 samples, respectively. All extraction and PCR controls for different DNA isolation, purification and amplification steps did not yield PCR products. In 35 PCR reactions we observed allelic dropout of 50% of heterozygous genotypes for HEL1, 60% for CSRM60, 75% for MM12, 73% for INRA5 and 64% averaged of the four loci, but never more than two alleles per marker in a given sample. The expected (He) and observed (Ho) heterozygosity values for a panel of European breeds calculated on the basis of 4 microsatellites ranged from 0.48 (Lagunaire breed) to 0.771 (Istrian breed) and from 0.43 (Lagunaire breed) to 0.76 (Istrian breed) respectively. The He and Ho values for pools of breeds ranged from 0.68 (Zebu breed pool) to 0.786 (Sicilian breed pool) and from 0.62 (Zebu breed pool) to 0.79 (Anatolian breed pool) respectively. These values were in the same range of those based on 30 microsatellites: He and Ho for panel of European breeds ranged from 0.544 (Lagunaire breed) to 0.741(Podolica breed) and from 0.51(Lagunaire breed) to 0.73 (Bohemian Red breed); He and Ho for pools of breed ranged from 0.67 (Zebu breed pool) and 0.780 (Anatolian breed pool) and from 0.59 (Zebu breed pool) and 0.72 (Busha breed pool) respectively (Table S1). The expected heterozygosity (0.698) in Ferento samples is within the range of the values found for modern breeds. We observed a clear heterozygote deficit (observed heterozygozity 0.588) related to allelic dropout for ancient samples.

Principal component analysis (Figure S1) as well as model-based clustering of Ferento cattle (not shown) combined with a panel of European breeds typed with the same markers differentiated three clusters: (1) African cattle, (2) Northwestern-European Lowland dairy breeds, and (3) Central-European and Iberian cattle as well as the ancient samples from Ferento. However, PCA did not establish relationships between Ferento cattle and individual breeds, since relative positions of breeds could not be reproduced with other panels of four microsatellites (not shown). We are aware that four microsatellites may have a low power in resolving diversity in populations that diverged since very few generations, however, we could not increase the number of markers that successfully and repeatedly amplified ancient DNA within the FAO list that permits to relate ancient to extant breeds.

**Figure 1:**
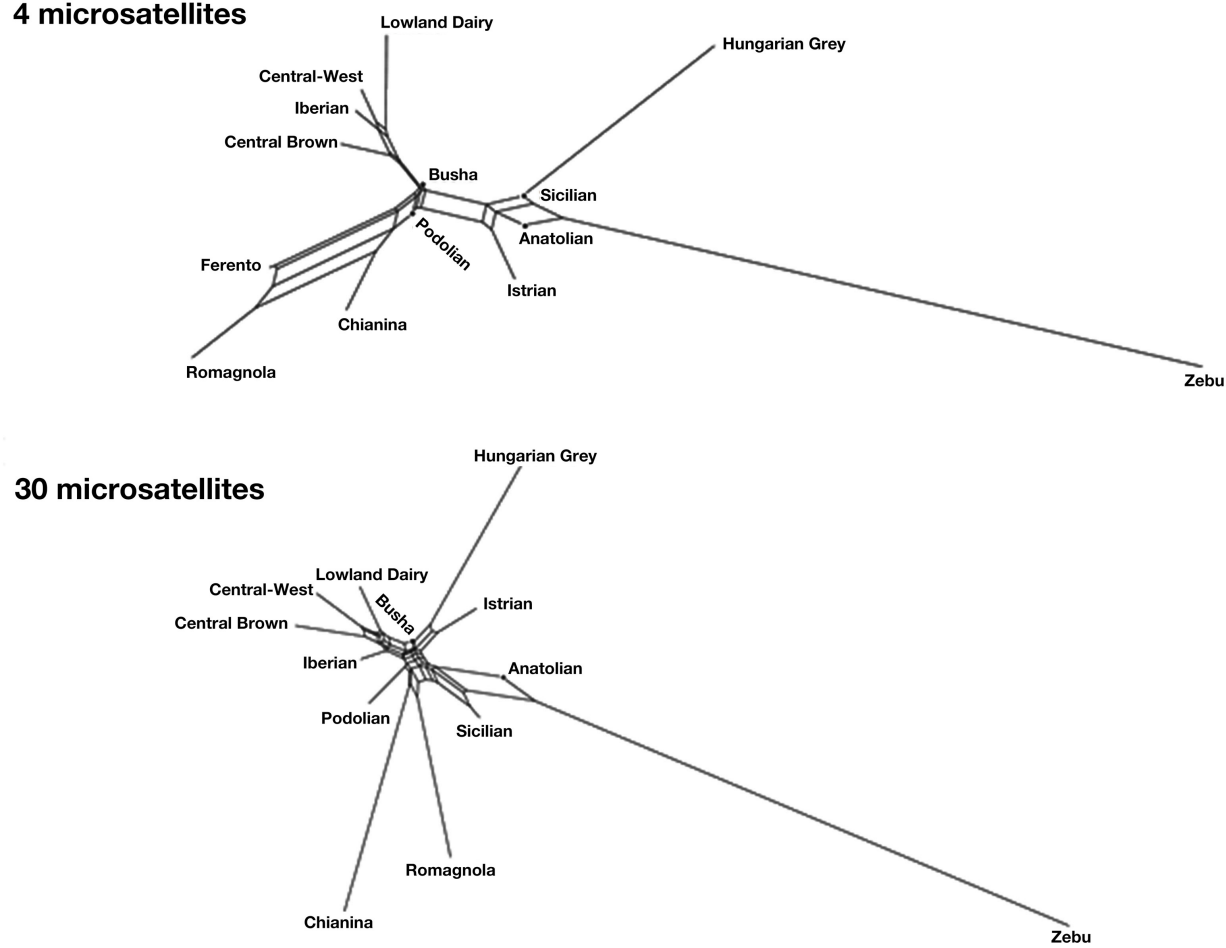
**NeighborNet graphs of Reynold's distances of Ferento cattle with modern Podolian breeds, and European, Anatolian and Indian breed clusters based on 4 or 30 microsatellites**. Ferento cattle is only shown with 4 microsatellites.

In order to get a more informative comparison of ancient Ferento genotypes with those of other cattle, we constructed NeighborNet graphs. Figure 1 shows NeighborNet graphs of the clusters with several modern Podolian breeds and ancient Ferento cattle. Although distances on the basis of only four microsatellites are not expected to be accurate, these distances reproduce the clustering that are generated by the 30 microsatellites. Interestingly, Ferento cattle is clearly linked with the Tuscanian Chianina and the related Romagnola cattle. The ancient and modern central Italian cattle is intermediate between (1) the Anatolian, Sicilian and Balkan Podolian breeds, and (2) the Iberian, Central European and Lowland dairy breeds. We observed that none of the individuals from modern breeds were as close to Ferento as the Chianina and Romagnola breeds.

## Discussion

In this study we demonstrate the feasibility of genotyping microsatellites for the study of cattle of 1000 years ago. The rates of amplification we have obtained (between 11 and 19%) appear to be lower than those found in in previous studies (Edwards et al., 2003; Nyström et al., 2012), which is probably due to relatively high temperature in central Italy. Although this limits a more elaborate analysis of the ancient genotypes, our results reconstruct the history of Italian cattle. The origin of the Podolian cattle in Italy is controversial (Felius et al., 2014). The first cattle arrived in the Italian peninsula at the start of the Neolithic (ca. 8000 BP). These long-horned cattle were largely replaced by short-horned cattle around 4500–5000 BP. Neolithic cattle gradually decreased in size and, as in the rest of Europe, Bronze and Iron age cattle in Italy had wither heights of typically 110 cm. However, cattle with wither heights of 115–135 cm were imported from Epirus in the centuries before the Roman era and are probably the ancestors of the large Roman cattle with wither heights of 150 cm or more (Kron, 2002). The analysis of mitochondrial DNA from modern taurine cattle has detected five mtDNA haplogroup with a specific geographic distribution (Lenstra et al., 2014). The relatively high frequencies of the T and T2 haplogroups of Italian Podolian cattle (Bonfiglio et al., 2010; Lenstra et al., 2014) may be explained by these imports of cattle. In an alternative scenario Anatolian cattle were brought to Tuscany by the founders of the Etruscan civilization (Pellecchia et al., 2007). After the Roman era, the large cattle disappeared from the European fossil record and cattle became even smaller than in the Iron age with a typical wither heights of 95 cm for the medieval Ferento cattle. Since the Renaissance cattle became larger and the Tuscan Chianina is now the largest cattle of the world. This breed and two other central-Italian breeds, Romagnola and Marchigiana, are related to the South Italian and Balkan long-hornd Podolian breeds (Felius, 1995). These cattle are believed to have emerged after the 12th century by local selection for long horns (Bökönyi, 1974). There have been several opportunities for gene flow between the Balkans and Italy, e.g., the consecutive migrations of Wisigoths, Osthrogoths and Lombards and the large scale import of Hungarian Gray cattle as “meat on the hoof” after the renaissance via Venice. The last imports have been well documented and involved not only oxes, but also fertile bulls (Appuhn, 2010). The deviating mtDNA haplogroup distribution of the Italian Podolian cattle indicates that any introgression of Balkan cattle was via bulls only (Lenstra et al., 2014). Alternatively, it has also been proposed that Italian Podolian cattle descends from ancient local cattle or even local aurochs (Ciani and Matassino, 2001).

The present data contributes to a historic reconstruction by indicating a link of the modern large, white and short-horned Chianina and Romagnola with local cattle living in the same area 1000 years earlier. This argues against an origin of the Chianina, Romagnola and the close relative Marchigiana from the postmedieval imports. Since the Germanic invasions are not likely to have replaced completely the local cattle, it is plausible that Ferento cattle descends from cattle kept during the Roman era. The large white central-Italian and Podolian cattle do not form a tight cluster in the network, which leaves open the possibility of an eastern origin of the long-horned Italian Podolian cattle. This may include the long-horned and feral Maremmana, for which no genotypes are available for all four microsatellites, but is in other networks close to the Hungarian Gray.

In conclusion our study demonstrates the feasibility and the potentiality of the analysis of nuclear DNA from ancient livestock remains.

## Author contributions

MG designed the study, carried out microsatellite amplifications experiments, and drafted the manuscript, LP contributed to the design of the work, JL contributed substantially to the analysis and interpretation of data and revised the work, ED provided ancient samples bones, ECGDC provided modern dataset, AV participated in developing ideas and revised the manuscript.

### Conflict of interest statement

The authors declare that the research was conducted in the absence of any commercial or financial relationships that could be construed as a potential conflict of interest.
