# Supplementary figures and images for: Microsatellite genotyping of medieval cattle from central Italy suggests an old origin of Chianina and Romagnola cattle
Source: Front Genet. 2015 Mar 4;6:68. doi: 10.3389/fgene.2015.00068 (PMC4349168; doi:10.3389/fgene.2015.00068)

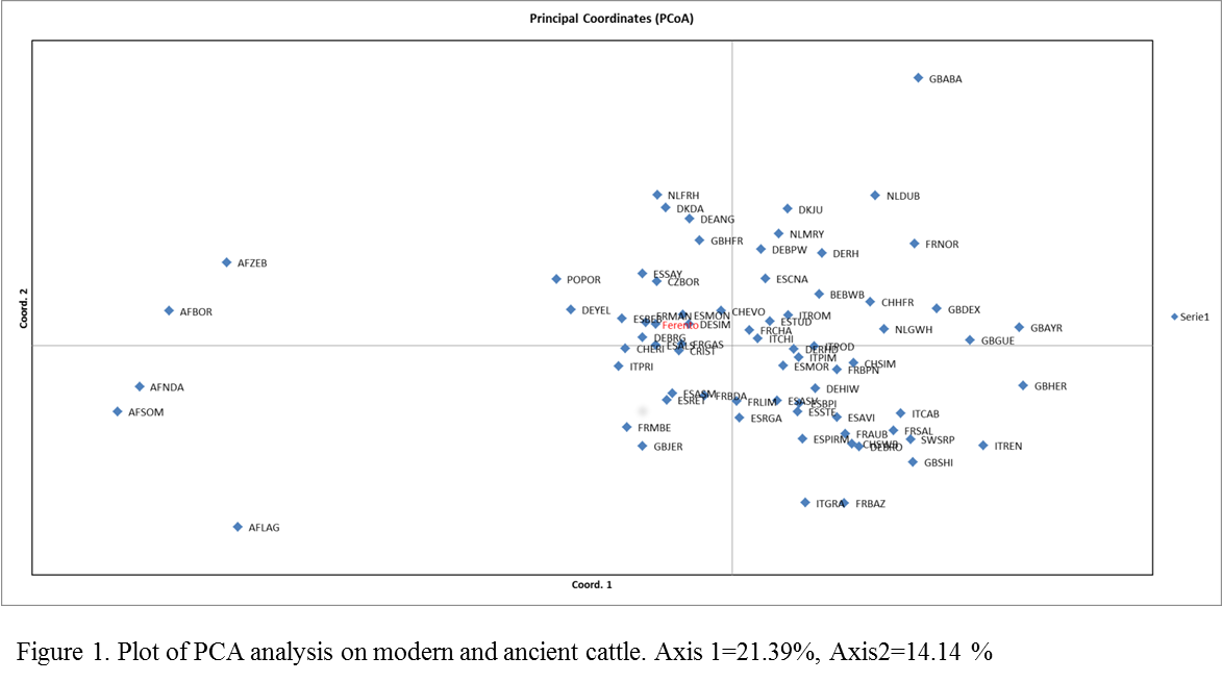

Supplement: Supplementary file 1 [file Image1.TIF]
