# Supplementary material for: Microsatellite genotyping of medieval cattle from central Italy suggests an old origin of Chianina and Romagnola cattle
Source: Front Genet. 2015 Mar 4;6:68. doi: 10.3389/fgene.2015.00068 (PMC4349168; doi:10.3389/fgene.2015.00068)
Supplement: Supplementary file 3 [file Table2.DOCX]

Table S2. Summary of microsatellite markers. BTA, bovine chromosome

| **Locus** | **BTA** | **Original allele** | **New allele** | **Annealing** | **Accession number** | **PCR primers (5'-3')** |  |  |
| --- | --- | --- | --- | --- | --- | --- | --- | --- |
|  |  | **size (bp)** | **size (bp)** | **temp (ºC)** |  |  |  |  |
| CSSM66 | 14 | 183 | 100 | 64 | … | GTGAGGAAGAAAGGGACAGGCA | | |
|  |  |  |  |  |  | GCCCAAGCTCCTCAGTGCATTA | | |
| ETH10 | 5 | 218 | 90 | 63 | Z22739 | TCTTCAGTGTAAGCAGTGGCTG | | |
|  |  |  |  |  |  | GAGCTCTGACGACACAGAGAAG | | |
| ETH152 | 5 | 199 | 111 | 63 | Z14040  G18414 | ACTGCTGTTGGCTTCCGGA | | |
|  |  |  |  |  |  | CAGACAGGTGCCCTCTGATCAC | | |
| ETH185 | 5 | 227 | 98 | 60 | Z14042 | TTGCAAAGAGTCGGACATGA | | |
|  |  |  |  |  |  | CAACTAAGACCTGATACAGCCAA | | |
| ETH225 | 9 | 158 | 96 | 61 | Z14043 | TCCAACATATGTGTGTGCATGC | | |
|  |  |  |  |  |  | AACTTCTCTAGTAGCAGCTGGC | | |
| HEL13 | 11 | 198 | 90 | 52 | X65207 | TTGGTGAGCTCTGAATGCTAGC | | |
|  |  |  |  |  |  | GAGGAAGCCAAGAAGGAGGGAA | | |
| HEL9 | 8 | 149 | 90 | 61 | X65214 | GCAGAGTTTGGCACATGCA | | |
|  |  |  |  |  |  | GTGGTGGTGAGAACATGGA | | |
| MM12 | 9 | 145 | 108 | 55 | Z30343 | CACAACATAATCACACATGCAA | | |
|  |  |  |  |  |  | TCGACTCTGGGGATGATGTA | | |
| ILSTS005 | 10 | 184 | 90 | 60 | L23481 | GGAAGCAATGAAATCTATAGCC | | |
|  |  |  |  |  |  | GCATCTGAAGTATGTATATACGGAAA | | |
| ILSTS006 | 7 | 290 | 104 | 63 | L23482 | TGGCACAATTCCATTCCTTT | | |
|  |  |  |  |  |  | GGAAGCGATCTAAACGTCCA | | |
| INRA063 | 18 | 177 | 116 | 60 | X71507 | CCCACAAAGTAACGACATAAATGTA | | |
|  |  |  |  |  |  | AAACTTTCTTCCAAGCATTTCTGTG | | |
| SPS115 | 15 | 247 | 118 | 59 | FJ828564 | GCTTCTCCAGAGCATCTCCA | | |
|  |  |  |  |  |  | AGATCCCATGTTGATCTGCTT | | |
| HEL1 | 15 | 104 | 104 | 55 | X65202 | CAACAGCTATTTAACAAGGA | | |
|  |  |  |  |  |  | AGGCTACAGTCCATGGGATT | | |
| CSRM60 | 10 | 99 | 99 | 61 | … | AAGATGTGATCCAAGAGAGAGGCA | | |
|  |  |  |  |  |  | AGGACCAGATCGTGAAAGGCATA | | |
| INRA005 | 12 | 139 | 139 | 55 | X63793 | CAATCTGCATGAAGTATAAATAT | | |
|  |  |  |  |  |  | CTTCAGGCATACCCTACACC | | |
