# Supplementary material for: Microsatellite genotyping of medieval cattle from central Italy suggests an old origin of Chianina and Romagnola cattle
Source: Front Genet. 2015 Mar 4;6:68. doi: 10.3389/fgene.2015.00068 (PMC4349168; doi:10.3389/fgene.2015.00068)
Supplement: Supplementary file 4 [file Table3.DOCX]

Table S3. Number of PCR and rate of successful amplifications for each sample and each locus. N (PCR), number of PCRs, N(+), number of successful amplifications

|  | **CSRM60** | |  | **HEL1** | |  | **INRA005** | | |  | **MM12** | |
| --- | --- | --- | --- | --- | --- | --- | --- | --- | --- | --- | --- | --- |
| **Code** | N (PCR) | N(+) |  | N (PCR) | N(+) |  | N (PCR) | | N(+) |  | N (PCR) | N(+) |
| **Ferento1** | 8 | 0 |  | 6 | 0 |  | 10 | 0 | |  | 5 | 0 |
| **Ferento2** | 9 | 6 |  | 6 | 3 |  | 15 | 4 | |  | 4 | 2 |
| **Ferento3** | 5 | 0 |  | 5 | 0 |  | 9 | 0 | |  | 5 | 0 |
| **Ferento4** | 9 | 2 |  | 5 | 0 |  | 14 | 0 | |  | 6 | 0 |
| **Ferento5** | 12 | 8 |  | 7 | 6 |  | 14 | 8 | |  | 3 | 2 |
| **Ferento6** | 7 | 3 |  | 6 | 3 |  | 12 | 5 | |  | 4 | 2 |
| **Ferento7** | 6 | 2 |  | 6 | 4 |  | 12 | 5 | |  | 4 | 2 |
| **Ferento8** | 7 | 0 |  | 5 | 0 |  | 12 | 0 | |  | 6 | 2 |
| **Ferento9** | 4 | 0 |  | 5 | 0 |  | 5 | 0 | |  | 5 | 0 |
| **Ferento10** | 5 | 0 |  | 5 | 1 |  | 6 | 0 | |  | 5 | 2 |
| **Ferento11** | 7 | 1 |  | 5 | 0 |  | 8 | 0 | |  | 4 | 0 |
| **Ferento 12** | 5 | 0 |  | 5 | 0 |  | 6 | 0 | |  | 5 | 0 |
| **Ferento 13** | 5 | 3 |  | 5 | 0 |  | 7 | 2 | |  | 5 | 2 |
| **Ferento 14** | 4 | 0 |  | 5 | 0 |  | 6 | 0 | |  | 5 | 0 |
| **Ferento 15** | 4 | 0 |  | 5 | 0 |  | 6 | 0 | |  | 5 | 2 |
| **Ferento 16** | 4 | 0 |  | 5 | 0 |  | 7 | 0 | |  | 4 | 0 |
| **Ferento17** | 4 | 0 |  | 5 | 0 |  | 6 | 0 | |  | 4 | 0 |
| **Ferento18** | 4 | 0 |  | 5 | 0 |  | 6 | 0 | |  | 4 | 2 |
| **Ferento19** | 9 | 0 |  | 5 | 0 |  | 6 | 0 | |  | 4 | 0 |
| **Ferento20** | 7 | 0 |  | 5 | 0 |  | 6 | 0 | |  | 4 | 2 |
| **Ferento21** | 15 | 3 |  | 5 | 0 |  | 15 | 0 | |  | 4 | 0 |
| **Ferento22** | 12 | 0 |  | 8 | 3 |  | 12 | 2 | |  | 6 | 0 |
| **Ferento23** | 12 | 5 |  | 7 | 2 |  | 10 | 0 | |  | 6 | 0 |
| **Ferento24** | 11 | 5 |  | 5 | 2 |  | 11 | 0 | |  | 6 | 0 |
| **Ferento25** | 11 | 0 |  | 4 | 0 |  | 11 | 0 | |  | 4 | 0 |
| **Ferento26** | 4 | 0 |  | 4 | 0 |  | 6 | 0 | |  | 4 | 0 |
| **Ferento27** | 4 | 0 |  | 4 | 0 |  | 6 | 0 | |  | 4 | 0 |
| **Ferento28** | 4 | 0 |  | 4 | 0 |  | 6 | 0 | |  | 4 | 0 |
| **Ferento29** | 4 | 0 |  | 4 | 0 |  | 6 | 0 | |  | 4 | 0 |
| **Ferento30** | 4 | 0 |  | 4 | 0 |  | 6 | 0 | |  | 4 | 0 |
| **Total** | 262 | 38 |  | 155 | 24 |  | 262 | 26 | |  | 137 | 24 |
